# Leprosy & gangrene: A rare association; role of anti phospholipid antibodies

**DOI:** 10.1186/1471-2334-5-74

**Published:** 2005-09-21

**Authors:** Shashank M Akerkar, Lata S Bichile

**Affiliations:** 1 Lecturer, Rheumatology,Dept of Medicine,Seth GSMC & KEM Hospital, Parel, Mumbai, India – 400012; 2 Chief Rheumatology,Head Dept of Medicine,Seth GSMC & KEM Hospital, Parel, Mumbai, India – 400012

## Abstract

**Background:**

Leprosy still remains an important public health problem for many parts of the world. An association of gangrene with leprosy is a rare one & can have a number of causative mechanisms. We present a case with Leprosy & gangrene with positive anti phopholipid antibody titers.

**Case presentation:**

A 50-year-old non-diabetic, non-hypertensive lady presented with 2 months history of progressive gangrene of bilateral toes. She was found to have madarosis & hypopigmented, hypoaesthetic macular lesions on the upper limb & thighs. Bilateral ulnar & popliteal nerves were thickened. A skin biopsy of the lesions revealed borderline tuberculoid leprosy, slit skin smears revealed a bacteriological index of 1+. She did not have any evidence of thromboembolic episode or atherosclerosis. ACLA was positive at presentation & also on another occasion 6 weeks later. ACLAs were of the IgM type on both occasions. Lupus Anticoagulant & β2 GPI antibody were negative. DOPPLER of the lower limb arteries did not reveal any abnormality. Patient was successfully treated with multi-drug antileprotics & anticoagulants.

**Conclusion:**

Infectious APLAs should be recognized as a cause of thrombosis in Leprosy. Appropriate anticoagulation can salvage limb function.

## Background

Leprosy still remains an important public health problem for many parts of the world. An association of gangrene with leprosy is a rare one & can have a number of causative mechanisms. We present a case with Leprosy & gangrene with positive anti phopholipid antibody titers.

## Case presentation

A 50-year-old non-diabetic, non-hypertensive lady presented with 2 months history of progressive blackish discoloration of the toes bilaterally. Examination revealed gangrene of the Right great toe, 2^nd ^toe & early gangrenous changes in the 3^rd ^toe. All the peripheral arteries were well felt, there was no radiofemoral delay. There was no cardiac murmur or a carotid bruit.

She was found to have madarosis & hypopigmented, hypoaesthetic macular lesions on the upper limb & thighs. Bilateral ulnar & popliteal nerves were thickened. A skin biopsy of the lesions revealed borderline tuberculoid leprosy. Slit skin smears revealed a bacteriological index of 1+. Erythrocyte sedimentation rate was 105, lipid profile & fasting sugars were normal & anti neutrophil cytoplsmic antibody (ANCA) negative.

Anti Cardiolipin antibody (ACLA) was positive at presentation (IgG-8; IgM-28.5; ELISA Genesis Diagnostics, Cambridgeshire, UK) & also on another occasion 6 weeks later (IgG-7.5; IgM-29; ELISA Genesis Diagnostics, Cambridgeshire, UK). Thus, ACLAs were of the IgM type on both occasions. Lupus Anticoagulant (PT, aPPT, Mixing studies, DRVVT) & β2 GPI antibody were negative (IgG-1; IgM-2.5; ELISA Genesis Diagnostics, Cambridgeshire, UK). DOPPLER of the lower limb arteries did not reveal any abnormality. Tests for other hypercoagulable states (protein C, protein S, Antithrombin III, homocystein, factor V Leiden) were normal.

The patient improved with the multi drug anti leprotics & anticoagulants. By 6 weeks, there was no progression of/ fresh gangrene & the pre gangrenous changes in the 3^rd ^toe had resolved.

## Discussion

Antiphospholipid antibodies (APLA) are a group of autoantibodies, which have been reported in Antiphospholipid syndrome (APS), which is characterized by raised levels of ACLA, thrombosis, recurrent fetal loss & thrombocytopenia. APLA is a generic term that describes closely related but not identical autoantibodies found in APS: ACLA, anti β2 GPI & those with lupus anticoagulant activity. The syndrome can occur in its primary form or secondarily in association with other autoimmune disorders. Although raised levels of these antibodies were first reported only in autoimmune diseases, their prevalence is now known to be more widespread. Elevated levels of these antibodies have been found in various infections like Syphillis, HIV disease, HCV disease, tuberculosis, cytomegalovirus infection [1]. Loizou et al studied 112 leprosy patients & found elevated titers of APLA in 29%, anti β2 GPI in 89%, & anti-Prothrombin in 21% of them [2]. Initially, it seemed that infection induced APLA are not associated with the thrombotic manifestations of APS. This was attributed to the fact that the binding of autoimmune APLA to phospholipid is enhanced by the cofactor β2 GPI (i.e. β2 GPI dependent) while the binding of infection induced APLA is not enhanced by this cofactor (i.e. β2 GPI independent). Recent studies, however show that the APLA in leprosy patients are heterogeneous with respect to their β2 GPI requirement: in 10 of 31 leprosy sera, the APLA were β2 GPI dependent & 16 of 31 were β2 GPI independent [3]. The clinical implications of this β2 GPI dependency are seen in Lucio's phenomenon in which the histopathological findings are related to microvascular thrombosis in the absence of inflammatory infiltration of the vessel wall. The β2 GPI dependency of APLA in this condition has been confirmed by Levi et al [4]. Apart from this evidence of microscopic thrombosis, frank gangrene in association with leprosy is a rare entity. It has been hypothesized that certain infections in genetically predisposed individuals may induce these APLA. Phospholipid binding peptides of bacterial & viral origin that have structural similarity to the phospholipid sites have been detected & found to induce APLA with properties similar to autoimmune APL in mice [5]. The elevated levels of IgM subtype of APLA seen in our patient is in accordance with other studies of APLA in leprosy [6,7].

Gangrene of the extremities in leprosy can have mechanisms other than APLA alone. Vascular changes in the form of intimal thickening & medial infiltration are known to occur in leprosy. Embolisation & resultant grafting of the Virchow cells has been found to lead to obstruction of the vessels [8]. Four such cases of arterial obstruction have been described; 2 of them being occlusion of the posterior tibial artery by lepromatous infiltration [9]. Arteriographic abnormalities such as occlusion, narrowing, tortuosity, dilatation, poststenotic dilatation, irregularity and incomplete filling of the lumen have been found in the digital circulation in more than 75–94% of leprosy patients [10].

Nerve trunk hypertrophy secondary to lepromatous process can lead to arterial entrapment in the osteoligamentous channels. This entrapment as well as the irritation of sympathetic fibers can lead to spasm of the vessels & resultant vascular compromise to the distal extremity. This has been confirmed with angiography & reversal of the spasm as well as the vascular compromise seen after release of the roof of the osteoligamentous channel [8].

Our patient did not have any clinical or laboratory markers of atherosclerosis or embolism, DOPPLER of the lower limbs did not reveal any vascular obstruction involving the medium size arteries. In the absence of any other hypercoagulable states, APLA remains the most probable cause of the digital gangrene.

## Conclusion

Infectious APLA should be recognized as a cause of thrombosis in Leprosy. Appropriate anticoagulation can salvage limb function. However, other mechanisms of gangrene need careful evaluation & appropriate management.

## List of abbreviations

APLA – anti phospholipid antibody

ACLA – anti cardiolipin antibody

β2 GPI – β2 Glycoprotein I

ANCA – Anti Neutrophil Cytoplasmic Antibody

APS – Anti Phospholipid Syndrome

PT – Prothrombin Time

aPPT – Activated Partial Thromboplastin Time

DRVVT – Dilute Russel's Viper Venom Time

## Competing interests

The author(s) declare that they have no competing interests.

## Authors' contributions

SA & LB carried out the study and conceived of the study. SA drafted the manuscript & LB reviewed the same. Both authors read and approved the final manuscript.

**Figure 1:**
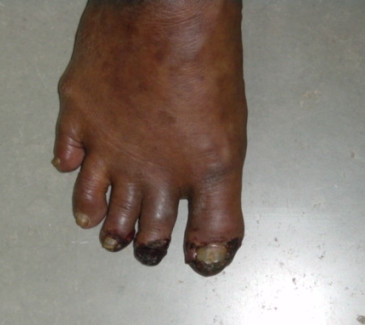
Gangrenous changes.

## Pre-publication history

The pre-publication history for this paper can be accessed here:


